# Interplay between Nanoparticles and Phosphorus Dendrimers, and Their Properties

**DOI:** 10.3390/molecules28155739

**Published:** 2023-07-29

**Authors:** Anne-Marie Caminade

**Affiliations:** 1 Laboratoire de Chimie de Coordination du CNRS, 205 Route de Narbonne, BP 44099, 31077 Toulouse CEDEX 4, France; anne-marie.caminade@lcc-toulouse.fr; 2 LCC-CNRS, Université de Toulouse, CNRS, 31077 Toulouse, France

**Keywords:** dendrimers, nanoparticles, hybrid materials, catalysis, sensors

## Abstract

This review presents the state of the art of interactions between two different families of nanoobjects: nanoparticles—mainly metal nanoparticles, and dendrimers—mainly phosphorhydrazone dendrimers (or dendrons). The review firstly presents the encapsulation/protection of existing nanoparticles (organic or metallic) by phosphorus-based dendrimers and dendrons. In the second part, several methods for the synthesis of metal nanoparticles, thanks to the dendrimer that acts as a template, are presented. The properties of the associations between dendrimers and nanoparticles are emphasized throughout the review. These properties mainly concern the elaboration of diverse types of hybrid materials, some of them being used as sensitive chemosensors or biosensors. Several examples concerning catalysis are also given, displaying in particular the efficient recovery and reuse of the catalytic entities.

## 1. Introduction

Nanoparticles are defined as matter that has at least one dimension between 1 and 100 nm, and that generally has different properties from its bulk. This is particularly the case for metal nanoparticles, such as gold nanoparticles (NPs), which have a different color depending on their size and differs from the color of bulk gold, in connection with their surface plasmon resonance [1]. Gold NPs are widely used for diagnostics and therapy [2]. Dendrimers are monodisperse hyperbranched polymers of nanometric size, very different from classical polymers, as they are synthesized step-by-step and not by bulk polymerization reactions [3]. Most dendrimers are synthesized by a divergent process, starting from a multifunctional core, possessing 2 to 8 functions in most cases. Such a divergent process frequently involves two steps. The first step is the modification of the core functionalities to allow them to react with a branched monomer, used in the second step. The branched monomers generally have either a 1 → 2 [4] or 1 → 3 branching motif [5], which allows the multiplication of the number of terminal functions by either two or three. The repetition of both steps—the modification of the terminal functions and the reaction with the branched monomer—induces the growth of the dendrimer size and the multiplication of the terminal functions. Each time the number of terminal functions is increased, a new “generation” is created. It is necessary to use only quantitative reactions for both steps, as it becomes rapidly impossible to separate a pure dendrimer from a dendrimer with one or a few missing branches. There is a theoretical limit for the growth of pure dendrimers [6], which depends essentially on the branch length. The highest generations reached were generation 11 [7] with polyamidoamine (PAMAM) dendrimers, created by Tomalia [8], generation 12 with polyphosphorhydrazone (PPH) dendrimers created by Caminade [9], and generation 13 with triazine dendrimers created by Simanek [10]. Another family of dendritic molecules are dendrons (dendritic wedges), which were first synthesized by convergent methods [11], but they can also be synthesized by divergent methods, such as dendrimers. The main difference between dendrimers and dendrons lies in the presence of one function at the core of dendrons, and not at the core of dendrimers. This single function can be used for the grafting of several dendrons to another core to produce a dendrimer [12], or for grafting a long alkyl chain for producing amphiphilic dendrons able to self-assemble [13].

**Figure 1:**
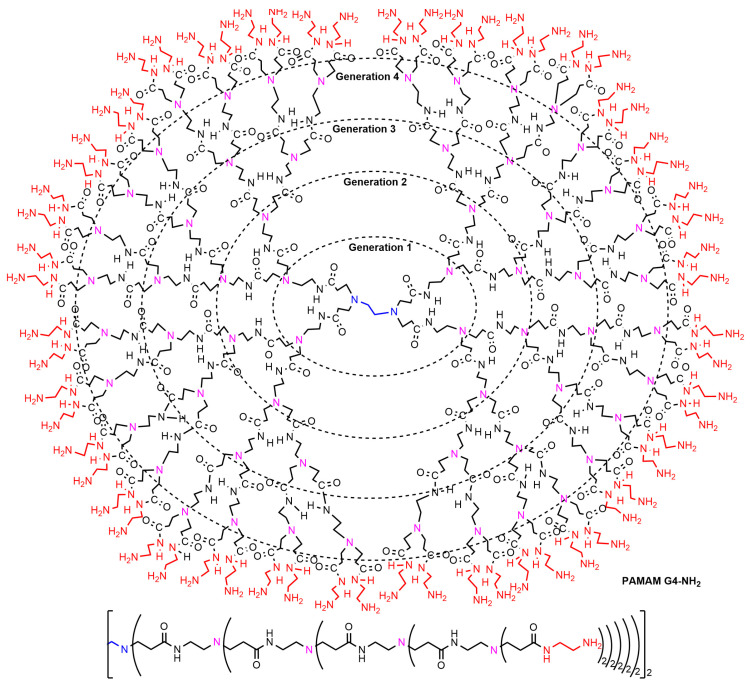
Fourth-generation PAMAM dendrimer having NH_2_ terminal functions [8]. Both the full (expanded) structure (each generation approximately encircled by a dotted oval) and the linear representation of the same dendrimer, with parentheses after each branching points, are represented.

Metal nanoparticles and dendrimers are hard and soft nanomaterials, respectively. They are both included in a theory proposed by Tomalia for unifying nanosciences and their complex relationships, directed accordingly by their analogous critical nanoscale design parameters (CNDPs). Such parameters concern (1) sizes, (2) shapes, (3) surface chemistries, (4) rigidity/flexibility, (5) architecture, and (6) elemental compositions [14]. Metal nanoparticles and dendrimers may have analogous sizes, shapes, and surface chemistries, but they have different rigidity/flexibility, architecture, and elemental composition. For these reasons, the interplay between both types of nanoobjects appears as a promising topic, which was studied for the first time by three different groups 25 years ago (in 1998) [15,16,17]. The three groups used PAMAM dendrimers as templates and hosts of metal nanoparticles. The structure of the fourth-generation PAMAM dendrimer is shown in Figure 1, with a different color for the core, the branching points of the different generations, and the surface functionalities. Two forms of the PAMAM dendrimer are represented in this figure, i.e., the developed structure, in which a dotted oval approximately encircles each generation, and the linear structure, with parentheses after each branching point, with a number indicating the number of branches emanating from each layer of branching points. This linear representation of dendrimers is used in most of the following figures and schemes. 

One of the three first examples of dendrimers interacting with nanoparticles was proposed by Zhao et al. [15]. They used hydroxyl-terminated fourth-generation PAMAM dendrimers (G4-OH) to trap up to 16 Cu^2+^ ions inside the structure, and the same generation terminated with amines (G4-NH_2_) to trap up to 36 Cu^2+^. It was proposed by the authors that, on average, each Cu^2+^ was coordinated to two primary amine groups on the surface. Chemical reduction with NaBH_4_ resulted in both cases in intra-dendrimer Cu clusters, having a diameter less than 1.8 nm with G4-OH, and >5 nm in diameter with G4-NH_2_. In the second example, Balogh et al. [16] used PAMAM G4-NH_2_ and PAMAM G5-NH(CH_2_)_2_NHCO(CH_3_)_3_ (pivalate) for the complexation of Cu^2+^, followed by the reduction using aqueous hydrazine, to afford nanoclusters of Cu. These clusters inside PAMAM dendrimers have been stored for more than 90 days at room temperature and found to be stable in the absence of oxygen. In the third example, Esumi et al. [17] described the preparation of Au colloids by the reduction of the metal salt HAuCl_4_ with UV irradiation in the presence of PAMAM-NH_2_ dendrimers of generations 0, 1, 2, 3, 4, and 5. The average particle sizes decreased with an increase in the number of surface amino groups of the PAMAM dendrimers. Au colloids with a diameter less than 1 nm were obtained in the presence of the largest dendrimers (G3–G5), indicating that the dendrimers have an effective protective action for the formed Au colloids. Indeed, particle growth would be prevented by the three-dimensional structure as the generation of the dendrimers increases, resulting in a smaller particle size.

After these pioneering works [15,16,17], many publications and different reviews have been published about the association of dendrimers with nanoparticles [18,19]. Different specialized fields of use have also been reviewed, in particular, catalysis [20,21,22] and biomedical applications [23], including bioimaging and drug delivery [24].

**Figure 2:**
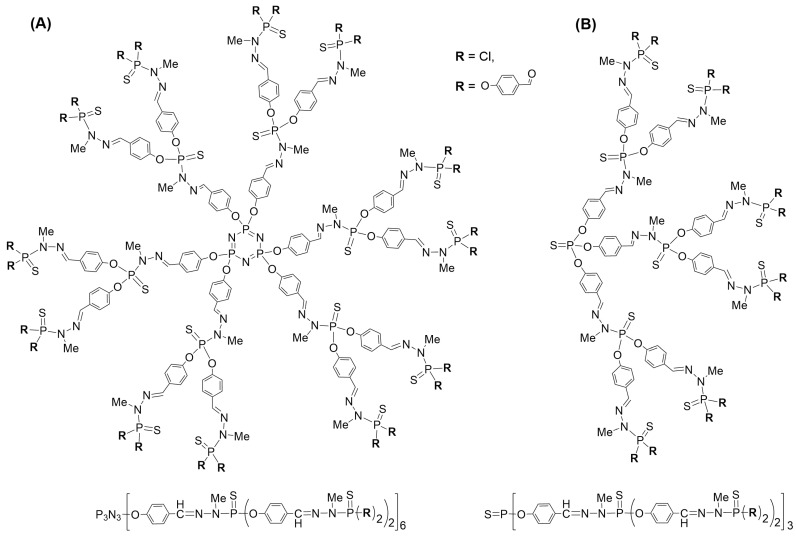
Polyphosphorhydrazone PPH dendrimers built from the hexafunctional core ((**A**) [26]) and the trifunctional core ((**B**) [25]). Both the full chemical structure and the linear structure with parentheses are represented for both compounds (generation 2 in both cases).

This review describes the use of a special class of dendrimers, phosphorus dendrimers of type polyphosphorhydrazone (PPH), for trapping or synthesizing nanoparticles. Such a type of PPH dendrimers is synthesized by a divergent process, using 4-hydroxybenzaldehyde as the linker and the phosphorhydrazide H_2_NNMe-P(S)Cl_2_ as the branched monomer. Two different cores have been essentially used, the trifunctional P(S)Cl_3_ [25] and the hexafunctional (P=N)_3_Cl_6_ [26]. Figure 2 displays the full structure and the linear structure of second-generation phosphorhydrazone dendrimers built from both types of cores. Such dendrimers are very modular [27] and display multiple properties, particularly in the fields of catalysis [28], materials [29], and biology/nanomedicine [30], for which it has been shown that the internal structure of the dendrimers plays a key role [31]. This review is organized depending on the role of the dendrimers, either the encapsulation/covering of existing nanoparticles, or the generation of the nanoparticles, and their protection.

## 2. Interaction between Phosphorus Dendrimers and Organic Nanoparticles

**Scheme 1:**
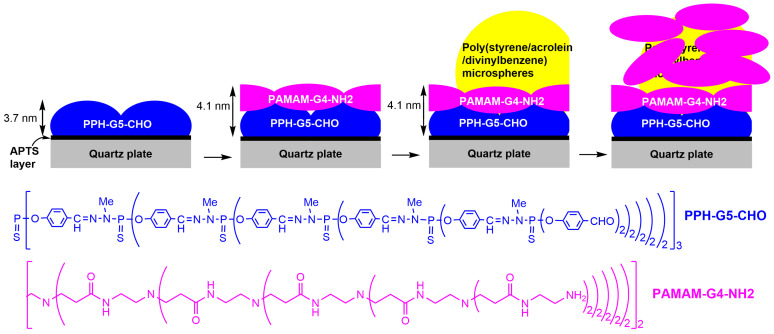
Structure of the 5th-generation phosphorus dendrimer, of the 4th-generation PAMAM dendrimer, and their use for the covalent decoration of a quartz plate, followed by the penetration of poly(styrene/acrolein/divinylbenzene) microspheres, and finally the deposition of a second layer of PAMAM-G4-NH2 dendrimers [32,33].

The very first example of phosphorus dendrimers interacting with (large) nanoparticles concerned organic particles consisting in poly(styrene/acrolein/divinylbenzene) microspheres with a narrow-diameter distribution (D = 300 nm). Quartz plates were sequentially modified with γ-aminopropyltriethoxysilane (APTS), followed by the covalent attachment of a single layer of the fifth-generation phosphorus PPH dendrimer, bearing aldehyde terminal functions. In a further step, a layer of PAMAM dendrimer of generation 4, bearing primary amine terminal functions, was covalently grafted to the layer of phosphorus dendrimers. Poly(styrene/acrolein/divinylbenzene) microspheres were then deposited and penetrated inside the soft layers of both types of dendrimers. In the last step, another layer of the fourth-generation PAMAM dendrimers was deposited. Surface layers were characterized through X-ray photoelectron spectroscopy (XPS) to determine the thickness of the layers and through contact angle measurements. The fabrication and the average thickness of each layer are reported in Scheme 1 [32]. In a related paper [33], the same plates were analyzed through atomic force microscopy (AFM) to measure the roughness of the surface of the modified plates, which displayed a uniform covering of the plates with the fifth-generation phosphorus dendrimer (PPH-G5-CHO) and with the second layer of the PAMAM dendrimer (PAMAM-G4-NH_2_).

**Scheme 2:**
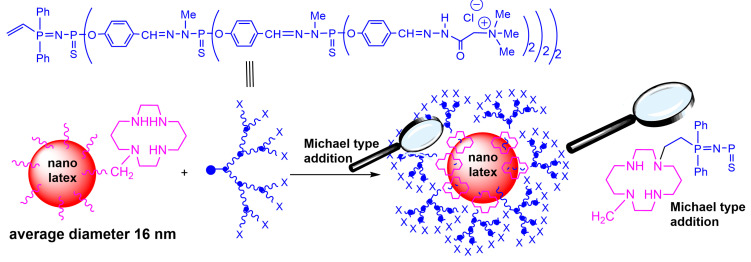
Decoration of a nanolatex with phosphorus dendrons, and structure of the second-generation dendron used [34]. The magnifying glass displays the result of the Michael reaction.

Latex nanoparticles, with an average diameter of 15 nm, were obtained by copolymerization of styrene and 4-vinylbenzyl chloride, followed by post functionalization with cyclam. These organic nanoparticles were reacted with phosphorus dendrons of different generations, having an activated vinyl group at the core and Girard’s reagent T (trimethylammonium) terminal functions. The average number of dendrons grafted per cyclam residue was 1 for generation 0, and approximately 0.7 and 0.4 for generations 1 and 2, respectively (Scheme 2). The dendronized nanolatexes at 2 to 4% in water formed translucent hydrogels upon standing at room temperature for one week. Approximately 105 × 10^3^, 185 × 10^3^, and 345 × 10^3^ water molecules were estimated to be gelled by each dendron of generations 0, 1, and 2, respectively [34]. The gelation is due to the terminal functions, as this gelation phenomenon was already observed with the same type of terminal functions on the surface of a dendrimer [35]. Indeed, electron micrographs of the dendrimer gels revealed a network of aggregated dendrimers trapping large quantities of water. Intermolecular hydrogen bonding, face-to-face π–π aromatic stacking, and hydrophobic effects of the internal backbone of the dendrimers were considered as the main factors explaining the creation of the network and the gelation properties [35].

## 3. Interaction of Dendrimers with Pre-Existing Metal Nanoparticles 

### 3.1. Gold Nanoparticles

Gold nanoparticles display a wide range of properties, depending on their size and the stabilizer used, and have been reviewed [36,37]. They have been used as catalysts [38]; as chemical and biological sensors [39]; and in medicine [40,41,42] for diagnosis, imaging, therapy [43], and drug delivery [44]. Two types of functionalized commercially available gold nanoparticles have been used in the interaction with phosphorus PPH dendrimers. A review has gathered the examples of the interplay of gold with phosphorus dendrimers, but focusing essentially on discrete complexes [45]. 

**Scheme 3:**
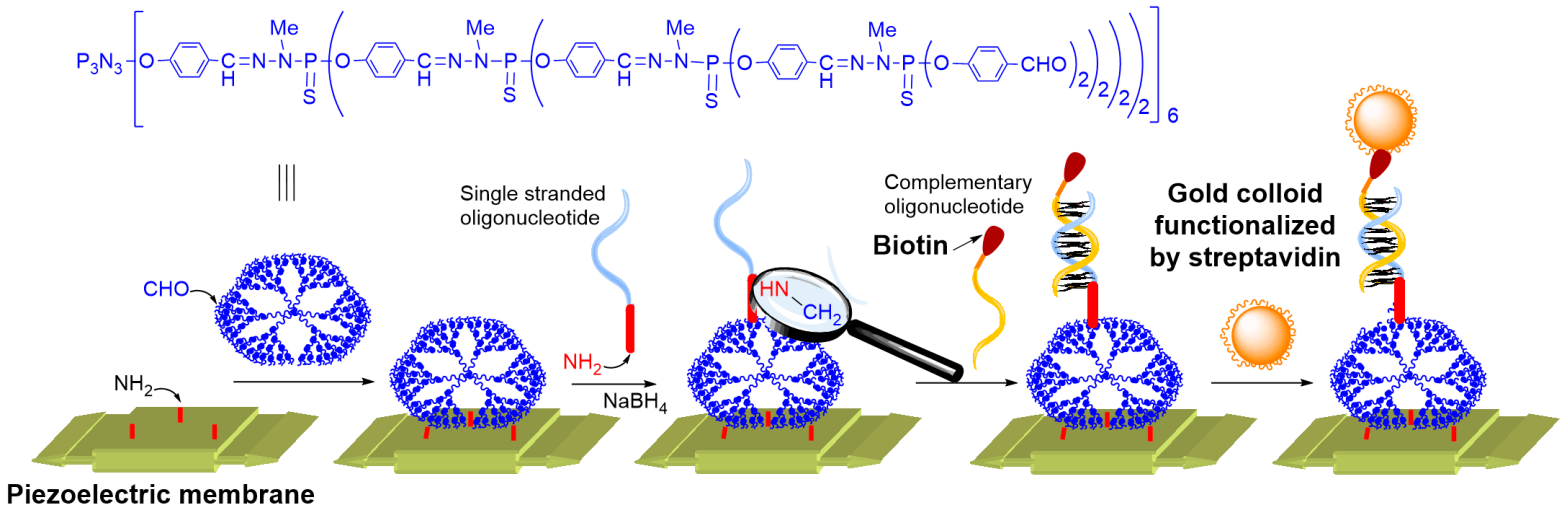
Step-by-step modification of a piezoelectric membrane for sensing gold nanoparticles [49]. Covalent bonding between dendrimer and oligonucleotide shown inside the magnifying glass.

In relation to the work shown in Scheme 1, a fourth-generation phosphorhydrazone dendrimer has been covalently grafted to a piezoelectric membrane, preliminarily functionalized with APTS; then, a single-stranded oligonucleotide functionalized on one end with a primary amine was covalently grafted, using a known procedure [46,47]. The resulting imine bonds were reduced with NaBH_4_ to afford stable secondary amines, and the remaining aldehydes on the surface of the dendrimers were reduced to alcohols. A complementary oligonucleotide functionalized on one end with biotin was then hybridized. The presence of dendrimers as 3D linkers between a solid surface and an oligonucleotide is known to favor the hybridization, as it occurs away from the solid surface, almost like in solution, contrarily to classical linkers that fall onto the surface [48]. This system was used as a very sensitive sensor for monitoring the real-time kinetics of the absorption of gold colloids functionalized with streptavidin (diameter 40 nm) on the functionalized membrane (Scheme 3) [49]. This method was chosen to profit from the well-known strong association of biotin with streptavidin [50,51,52]. The mass sensitivity of the device, estimated as −3.9 Hz/pg, was better than state-of-the-art values for piezoelectric mass-sensing devices by a factor of several hundred at that time (2005) [49].

**Scheme 4:**
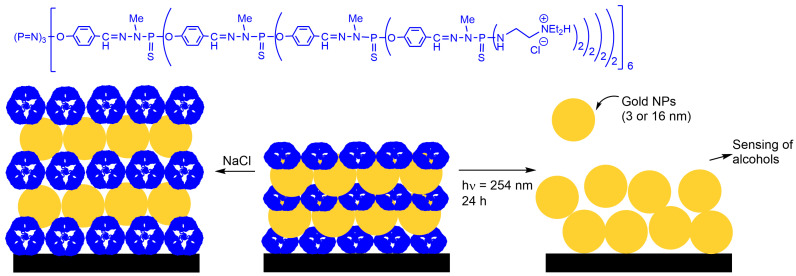
Structure of fourth-generation dendrimer functionalized with ammoniums on its surface, and its use for the layer-by-layer elaboration of a multilayer with negatively charged gold nanoparticles [56].

Besides the covalent grafting of dendrimers to the surface shown in the previous paragraphs, they can also be deposited layer-by-layer by electrostatic interactions on positively charged surfaces. Such positive surfaces were obtained by first coating the surface with 3-(diethoxymethylsilyl) propylamine (3-APDMES). Using alternate negatively and positively charged dendrimers, both of generation 4, enabled the step-by-step increase in the thickness of the coating by electrostatic interactions [53]. An analogous process was carried out with anionic gold nanoparticles (Au NPs), instead of the anionic dendrimers. These Au NPs equipped with anionic carboxyl functional groups were synthesized according to published procedures [54,55], and had diameters of approximately either 3 or 16 nm, as determined by TEM (transmission electron microscopy). These Au NPs and fourth-generation cationic phosphorus dendrimers (48 ammonium terminal functions) were deposited alternately on 3-APDMES-coated substrates (silicon wafer or quartz of a micro-balance) [56]. The deposition was carried out by immersing the substrates into Au NP colloidal suspensions for several minutes, followed by a rinsing step to remove any physically adsorbed NPs, and blow-drying with nitrogen. The same process was also carried out with the positively charged dendrimers, and each step was monitored by UV-vis spectroscopy. Up to 10 bilayers (Au NPs/dendrimers) were deposited. Upon addition of salt (NaCl), the dendrimers were swollen, probably due to reduced electrostatic repulsion between neighboring identically charged species [57], leading to an increase in the average distance between Au layers. The films were also exposed to deep UV light with a wavelength of 254 nm for 24 h, with the aim of removing the dendrimers (Scheme 4). Indeed, the dendrimer strongly absorbs at this wavelength and is destroyed by prolonged irradiation, as already observed in MALDI-Tof spectrometry, which used a UV-laser for desorption [58]. These films composed of Au nanoparticles (and a few remaining dendritic residues) were investigated in the sensing of five alcohols with different refractive indices, which were methanol, ethanol, 1-propanol, 1-butanol, and 1-pentanol, whose refractive indices are 1.329, 1.361, 1.385, 1.399, and 1.410, respectively. The absorbance peak (λ_max_) of Au multilayer films exhibited a moderate red-shift as the refractive index of the solvents increased, showing their potential as chemical sensors [56].

### 3.2. ZnCdSe Quantum Dots

**Figure 3:**
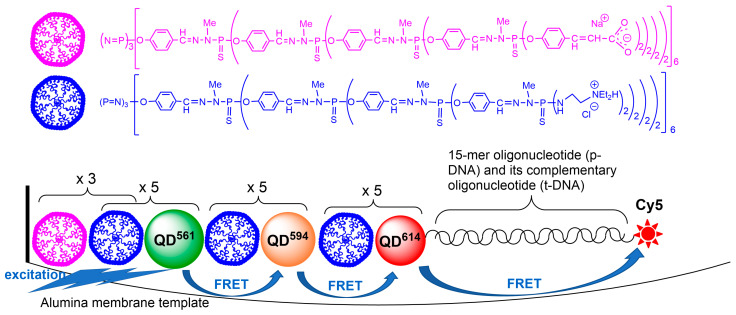
Schematization of a device comprising charged dendrimers and quantum dots associated with electrostatic interactions, for the sensitive detection of DNA [59].

Based on the method shown in Scheme 4, layer-by-layer electrostatic deposition inside the porous alumina membrane, of 400 nm in diameter with a pore depth of 80 μm, was carried out with positively and negatively charged phosphorus dendrimers. After removal of the template, such a process afforded nanotubes made of dendrimers [53]. The same process inside porous alumina was applied to quantum dots (QDs), which are fluorophores based on metallic nanoparticles such as ZnCdSe alloys, instead of the negatively charged dendrimers. In fact, three bilayers of positively and negatively charged dendrimers were first deposited inside the pores, previously functionalized with 3-APDMES (Figure 3). Five bilayers of positively charged dendrimers and negatively charged QDs with a luminescence maximum at *λ* = 561 nm (QD^561^, green) were then deposited, followed by five bilayers of the same type, based on QD^594^ (orange), and finally five analogous bilayers based on QD^614^ (red). A graded-bandgap was observed with these nanotubes composed of dendrimers and quantum dots, as they exclusively show an emission peak centered at *λ* = 614 nm, originating from QD^614^. On the top of this perfectly controlled multilayer, a 15-mer probe DNA (p-DNA) was immobilized and used for hybridization with Cy5-labeled complementary DNA (t-DNA) (Figure 3). Detection through fluorescence/Förster resonance energy transfer (FRET) displayed an increase in the Cy5 emission, which received an energy transfer of approximately 3.2% from the quantum dots. Although the energy transfer efficiency from the QDs to Cy5 is relatively low, it is sufficient to ensure the sensitive detection of DNA hybridization, with an enhancement factor of, ca. 15, indicating that such devices have potential utility for the detection of trace amounts of DNA [59]. Such devices were also elaborated on Au-coated glass surfaces, but not tested for DNA hybridization [60]. The work concerning the layer-by-layer modification of materials with dendrimers has been reviewed [61].

### 3.3. Cobalt Nanoparticles

**Scheme 5:**
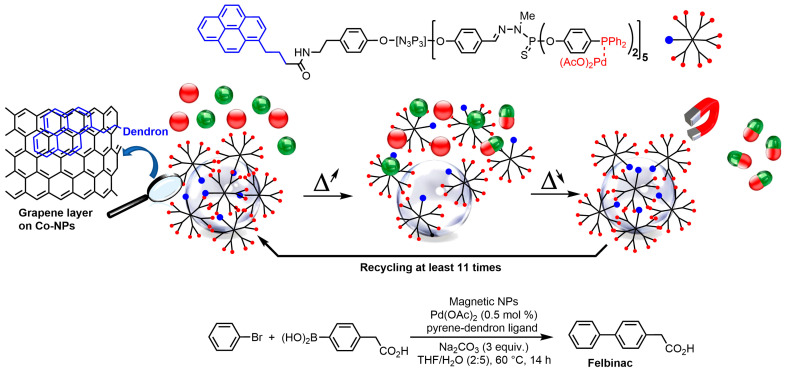
Cobalt nanoparticle covered by a few layers of graphene, and by dendrons interacting by π-stacking (see the magnifying glass), used as easily recovered catalyst in Suzuki reactions [62].

Cobalt nanoparticles covered by a few layers of graphene were used as support for catalysts [62]. Cobalt nanoparticles (Co NPs) are magnetic; thus, they can be easily recovered using a magnet [63]. In the first experiment, two phosphorus dendrons (generations 0 and 1) bearing a pyrene at the core and 5 or 10 triphenyl phosphines as terminal functions were used for complexing palladium, as previously reported [64]. The specific reaction of one of the chlorides of the cyclotriphosphazene N_3_P_3_Cl_6_, affording one function different from the five others, has been used for the synthesis of these dendrons. Such a possibility was previously used for the synthesis of dendritic structures, and has been reviewed [65]. The dendrons shown in Scheme 5 cover the Co-NPs at room temperature by π-stacking interactions between the pyrene at the core of the dendrons and the graphene layers on the NPs. Heating in solution fragilizes this interaction, and the dendrons go inside the solution, where they can perform catalysis. At the end of catalysis, cooling the reaction medium permits the dendrons to interact again by π-stacking with the NPs, which can be taken off the reaction medium using a magnet. In this way, the dendron can be easily recovered and reused. The concept was applied to the synthesis of Felbinac (an anti-inflammatory drug) by Suzuki coupling [66], using the zeroth-generation dendron (five phosphine complexes) (Scheme 5). The catalytic dendrons were recovered eleven times, displaying even at run 12 a 100% yield in Felbinac [62]. 

**Scheme 6:**
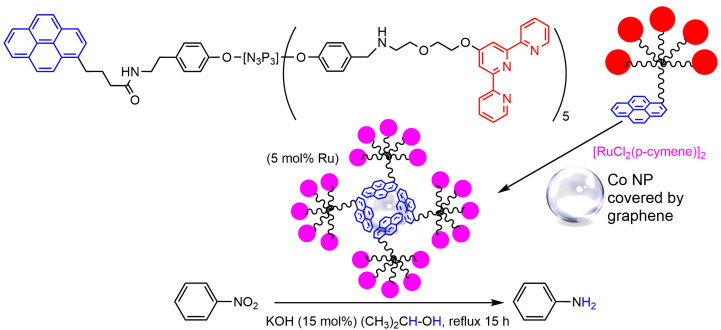
Transfer hydrogenation catalysis with dendrons interacting by π-stacking with Co-NPs [67].

A related work was carried out with a small dendron functionalized with a pyrene at the core and five terpyridines on the surface, suitable for the complexation of ruthenium. This complex was used for catalyzing the hydrogenation of nitrobenzene to aniline, using 2-propanol as the transfer hydrogen source (Scheme 6). In that case also, the dendron onto the cobalt NPs could be recovered using a magnet and reused at least seven times [67]. The efficiency of magnetic nanoparticles as versatile supports for catalysts has been reviewed [68].

**Figure 4:**
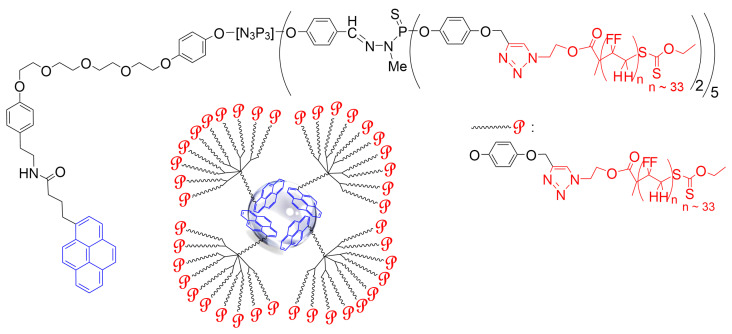
Dendron functionalized with 10 poly(vinylidene fluoride) chains, and interacting by π-stacking with Co-NPs [69].

The same type of π-stacking interaction was also applied to a dendron bearing a pyrene moiety at the core and 10 poly(vinylidene fluoride) (PVDF) chains on the surface. In order to maximize the efficiency of interaction with the Co-NPs, a long linker was used between the pyrene and the cyclotriphosphazene (Figure 4). The stability of the π-stacking interactions when the temperature increased and the reversibility of the process when the temperature decreased were studied. A partial release of the dendron from the surface of the Co-NPs was observed at 60 °C, but a partial reversibility to the π-stacking was observed on cooling to 20 °C. [69].

## 4. Dendrimers for the Synthesis of Metal Nanoparticles

All the above-mentioned experiments concerned the use of commercially available or pre-existing nanoparticles. However, poly(phosphorhydrazone) dendrimers are also suitable templates for the synthesis of diverse metal nanoparticles. 

### 4.1. Synthesis of Gold and Silver Nanoparticles

**Scheme 7:**
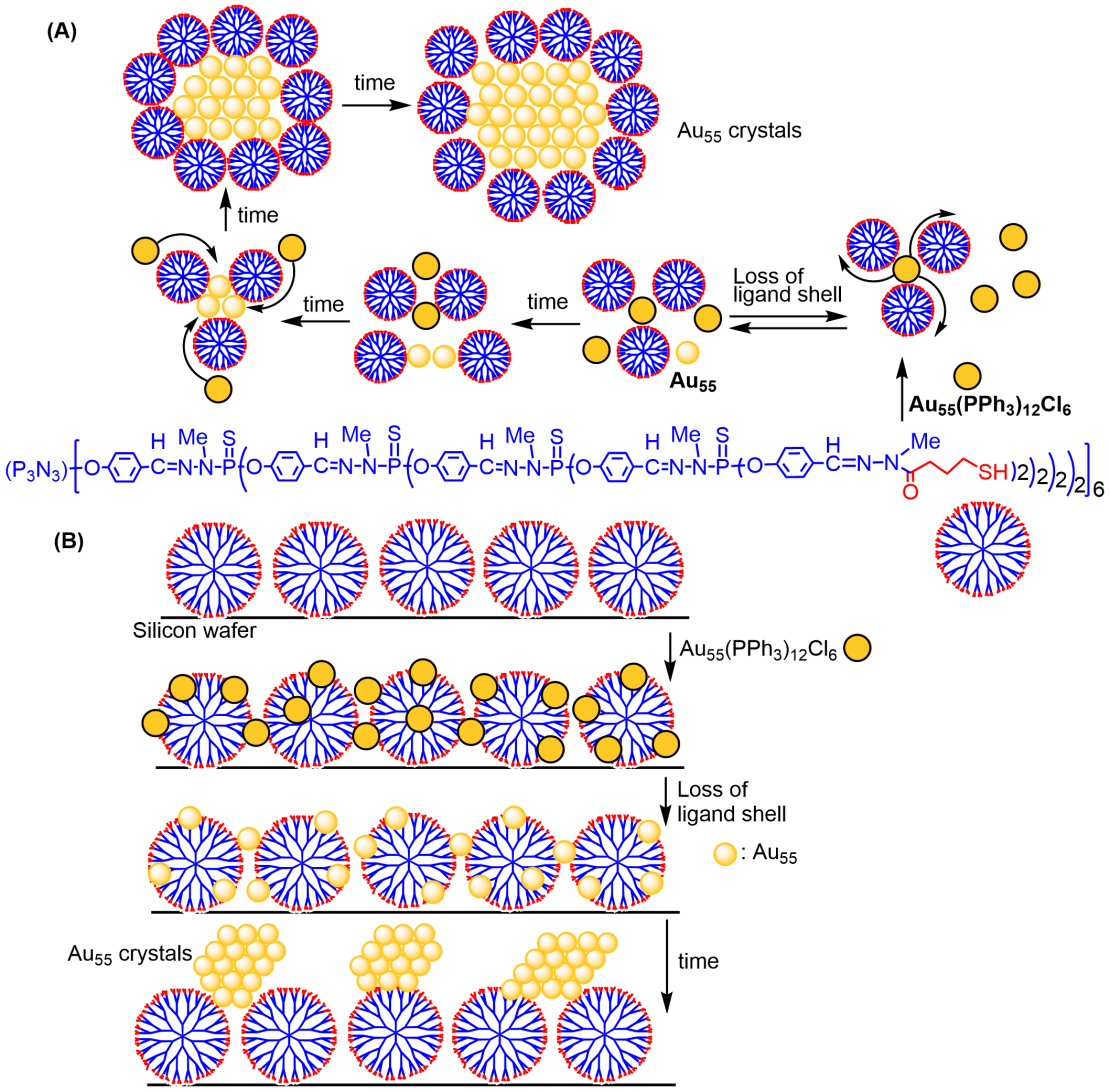
Structure of the thiol-terminated dendrimers and their application for the synthesis of Au_55_ nanocrystals, either in solution (**A**) [71] or on a silicon wafer (**B**) [72].

The first example with phosphorus dendrimers concerned a thiol-terminated fourth-generation dendrimer, equipped with 96 thiol (SH) functions, interacting with the gold cluster Au_55_(PPh_3_)_12_Cl_6_ obtained by reduction of PPh_3_AuCI in benzene at 50 °C by B_2_H_6_ [70]. The first experiments were carried out in solution in dichloromethane, and resulted for the first time in the formation of bare Au_55_ clusters, which self-associated in microcrystals of (Au_55_)_∞_ [71]. The dendrimer played two roles in this experiment: first, it removed the triphenylphosphine and chlorine ligands; then, it acted as a template for growing the crystals of Au_55_, as illustrated in Scheme 7A. Characterization by transmission electron microscopy (TEM), small-angle X-ray diffraction (SAXRD), wide-angle X-ray diffraction (WAXRD), X-ray absorption fine structure (EXAFS), energy-dispersive X-ray spectroscopy (EDX), and IR analyses confirmed the absence of ligands, and the preservation of the Au_55_ cluster structure in the crystals [71].

The same thiol-terminated dendrimer was then used in an analogous experiment but carried out in the solid state. A solution of dendrimer in dichloromethane was deposited on a 9 × 9 mm silicon wafer fixed onto a spin-coater, rotating at 100 rpm, to produce a single layer of dendrimers. The wafer was then washed with dichloromethane to eliminate the unbound dendrimers. Analysis by atomic force microscopy (AFM) indicated a thickness of 1.5–2.0 nm of the almost-defect-free layer. Interaction with the gold clusters was carried out by dipping the wafer platelet for a short time into a solution of the gold clusters. After washing with dichloromethane and drying, if the wafer was kept in an atmosphere of dichloromethane for about one week, nanosized crystals of bare Au_55_ clusters were observed on the dendrimer surface, as illustrated in Scheme 7B. This process taking place in two-dimensional reactant arrays is analogous to the process observed in three dimensions in solution [72].

**Scheme 8:**
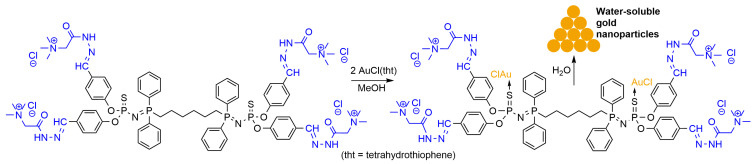
Elaboration of gold NPs using a small dendrimer Au complex in water [75].

A small water-soluble phosphorus-containing dendrimer, functionalized with four Girard’s reagents T (acethydrazide trimethylammonium chloride) and two P=N-P=S linkages, was especially synthesized for obtaining gold crystals from AuCl(tht) (tht = tetrahydrothiophene). AuCl(tht) was synthesized by the reaction of tht with HAuCl_4_ in water/methanol [73]. This dendrimer was especially engineered for reacting with AuCl(tht). Indeed, it was previously shown that the sulfur atom included in P=N-P=S linkages is specifically suitable for the complexation of AuCl, whereas the other P=S groups included in the dendrimers, but not in P=N-P=S linkages, do not react [74]. In addition, a reaction was rapidly observed at room temperature in water between Girard’s reagent T and AuCl(tht), leading to a black precipitate, showing that this reagent is indeed able to reduce gold, but not in the form of nanoparticles. With the hydrazone bond being slightly in equilibrium with the aldehyde and hydrazine forms, the dendrimer functionalized with this reagent should release a small quantity of Girard’s reagent T in water. This dendrimer acted first as a complexing agent for AuCl. The complex was stable in many types of solvents, but when adding water, the small quantity of released Girard’s reagent T acted as a mild reducer, and the structure of the dendrimer was finally a template for the formation of gold nanocrystals (Scheme 8). Shape-controlled Au nanoparticles in the form of triangles and associated triangles were obtained [75].

**Scheme 9:**
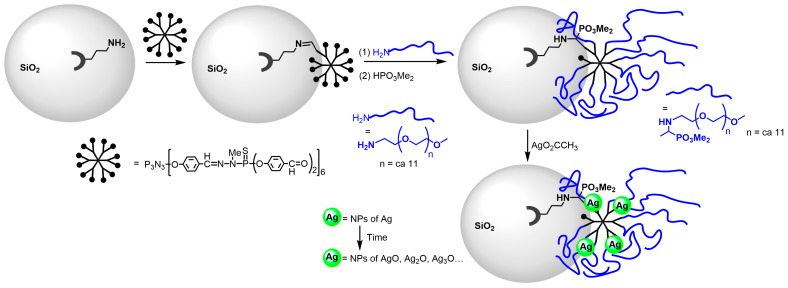
Silica NPs functionalized with pegylated dendrimers, suitable for hosting silver NPs, which display anti-bacterial properties [77].

Besides gold, silver nanoparticles were also obtained from phosphorus dendrimers. In the first step, a first-generation dendrimer functionalized with aldehydes was grafted to silica nanoparticles (mean diameter 12 nm), bearing amine surface functions issued from aminopropyltriethoxysilane (APTES) [76]. In the second step, polyethylene glycol (ca., 11 CH_2_CH_2_O units) having an amine at one end was reacted with the remaining aldehyde functions of the grafted dendrimers. To stabilize the imine functions, a hydrophosphorylation with dimethyl phosphite was carried out, producing a secondary amine and a dimethyl phosphite in place of all the imine groups. The reaction was in particular characterized by IR, which showed the disappearance of the aldehyde functions. The addition of silver acetate in a water suspension of the modified silica induced the darkening of the suspension, inducing the formation of silver nanoparticles on the surface of silica, both with and without a reducing agent (NaBH_4_) (Scheme 9) [77]. A typical plasmon absorption band centered between 394 and 416 nm was observed only when using the reducing agent. Evolution with time produced various silver oxide nanoparticles (AgO, Ag_2_O, Ag_3_O…). The antibacterial properties of silver NPs are well known [78,79]; thus, the properties of these materials were estimated by determining the minimal inhibitory concentration (MIC) and the minimal bactericidal concentration (MBC) on four typical bacterial strains (Gram+: *S. aureus* and *E. hirae*; Gram−: *E. coli* and *P. aeruginosa*). The silver-loaded silica NPs exhibited bacteriostatic activities against all bacterial strains in the 25–500 ppm range (silver equivalents), as shown in Table 1 [77]. 

### 4.2. Synthesis of Palladium, Platinum, and Ruthenium Nanoparticles

**Scheme 10:**
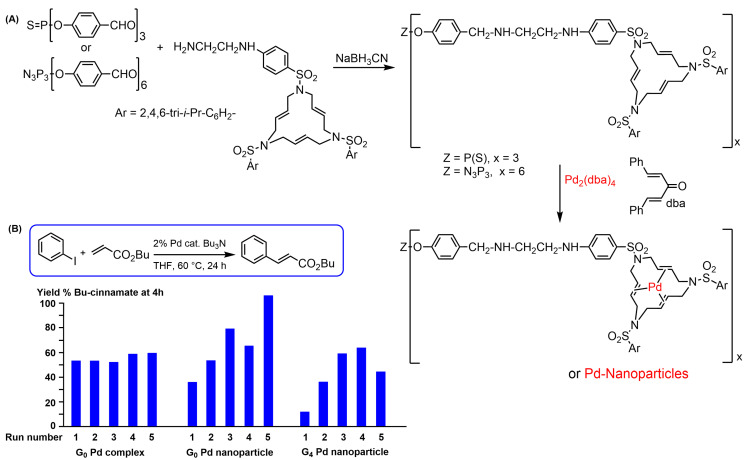
(**A**) Diagram of 15-membered macrocycles on the surface of dendrimers, used for the complexation of Pd and of Pd NPs [80]. (**B**) Both types of materials were suitable catalysts in Mizoroki–Heck reactions. The run numbers indicate the number of recovery and reuse cycles of the catalysts.

Triaza triolefinic 15-membered macrocycles functionalized with a diamine (Scheme 10) were reacted with the aldehyde terminal functions of generations 0, 1, and 4 of phosphorhydrazone dendrimers, followed by the reduction of the imine bonds with NaBH_3_CN to obtain stable compounds. Reaction with the palladium complex Pd_2_(dba)_4_ (dba = dibenzylidene acetone) afforded discrete complexes when using a strict stoichiometry of reagents (one Pd per macrocycle), or Pd nanoparticles when using an excess (Scheme 10A) [80]. Both the discrete complexes and the Pd NPs stabilized by the dendrimers were used as catalysts in Mizoroki–Heck reactions, monitored by ^1^H NMR [81]. Both types of catalysts could be recovered and reused several times. The catalytic efficiency of the discrete complexes did not change with the number of reuses (four times), whereas the nanoparticles became more and more efficient with the number of reuses, particularly when stabilized with the zeroth-generation dendrimer (Scheme 10B). This result can be explained by a decrease in the size of the nanoparticles with recycling, as they were less stabilized by the small zeroth-generation dendrimer than by the large fourth-generation dendrimer [80].

**Figure 5:**
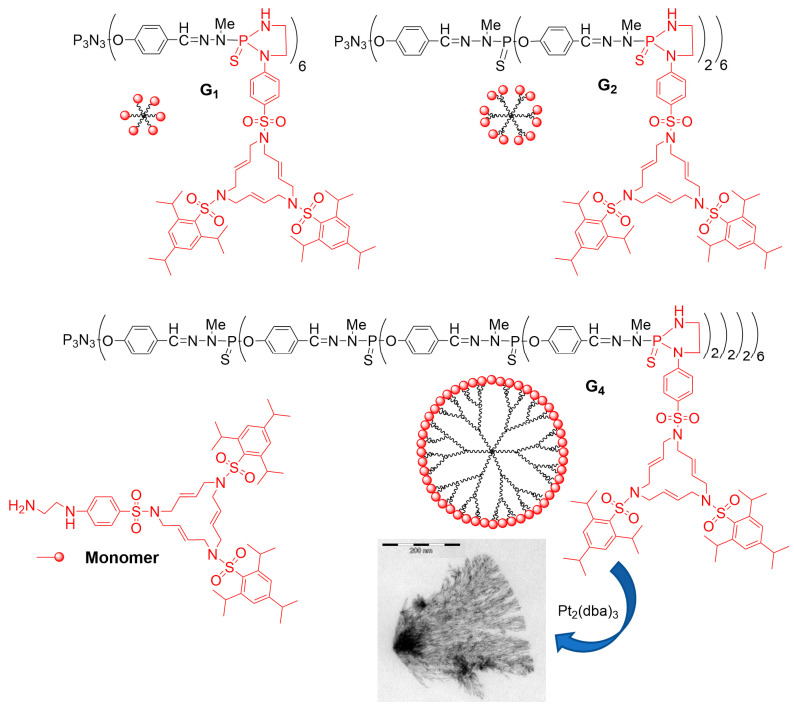
Diagram of 15-membered macrocycles grafted on the surface of phosphorhydrazone dendrimers, forming 5-membered heterocycles. Reaction with Pt_2_(dba)_3_ afforded dendritic networks of Pt nanoparticles (image) [83,84].

The same type of macrocycles was also grafted to the P(S)Cl_2_ terminal functions of phosphorhydrazone dendrimers (Figure 2, R = Cl). Two Cl were needed for grafting one macrocycle, by the formation of a five-membered phosphadiaza heterocycle. A monomer and generations 1, 2, and 4 were synthesized (Figure 5), and they were reacted with the platinum complex Pt_2_(dba)_3_ [82]. Platinum nanoparticles were obtained in all cases, which organized in dendritic networks when using the dendrimers. The mean length of the dendritic branches composed of Pt NPs surprisingly increased with the generation of the dendrimers, with the longest branches being obtained with generation 4, as shown by the image in Figure 5, obtained using high-resolution transmission electron microscopy (HRTEM). No network was observed when using the monomer. It was proposed that large dendrimers having numerous macrocycles as terminal groups should wrap Pt NPs more efficiently and produce longer ribbons than the smaller ones [83,84]. 

**Scheme 11:**
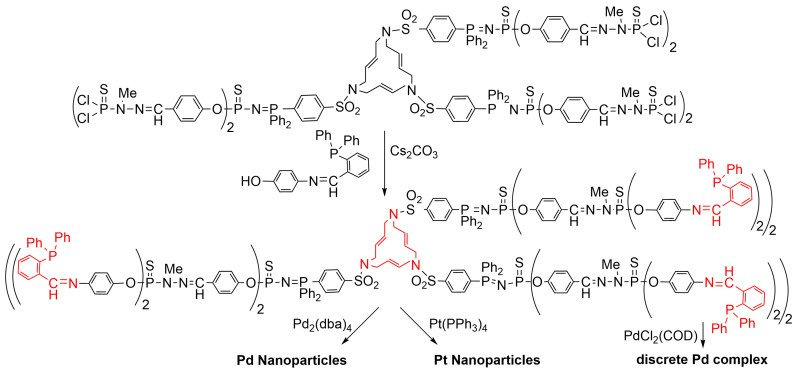
Dendrimers built from a 15-membered macrocycle core and functionalized with iminophosphines on the surface, forming discrete Pd complexes on the surface by reaction with PdCl_2_(COD), and Pd or Pt nanoparticles, by reaction with Pd_2_(dba)_4_ or Pt(PPh_3_)_4_, respectively [85].

Another family of phosphorhydrazone dendrimers was built from the same type of 15-membered triazatriolefinic macrocycle, which was used as the core instead of as terminal functions [85]. The dendrimers were built up to generation 3 and were functionalized on the P(S)Cl_2_ terminal functions with a phenol bearing an iminophosphine, at the level of the second and third generations (Scheme 11). The phosphines on the surface were suitable for the complexation of palladium dichloride PdCl_2_ from PdCl_2_(COD) (COD: cyclooctadiene), as shown previously [86], but not the macrocycle at the core. Indeed, these macrocycles are suitable for the complexation of Pd^0^, but not Pd^II^ [87]. However, the single macrocycle at the core of these dendrimers was suitable for the complexation of Pd^0^, and for the elaboration of palladium nanoparticles from Pd_2_(dba)_4_ and of platinum nanoparticles from Pt(PPh_3_)_4_, using an excess of metal in both cases. It was shown in these latter cases that the phosphines on the surface were not involved in the complexation of the nanoparticles [85].

**Scheme 12:**
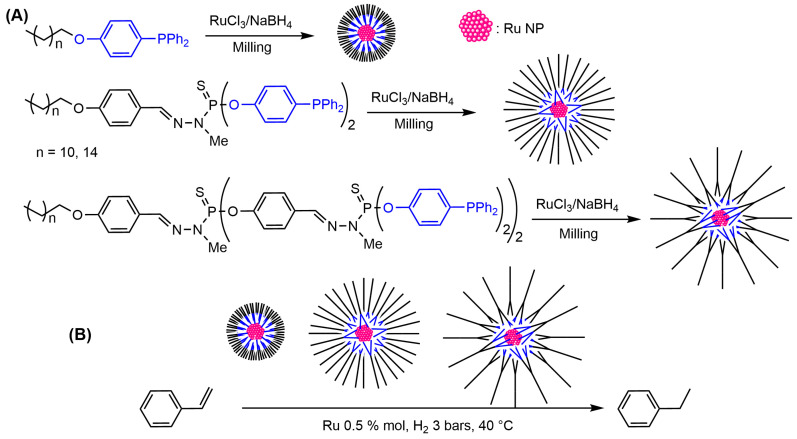
(**A**) Structure of dendrons having phosphines on their surface, and their use for obtaining Ru nanoparticles. (**B**) Hydrogenation of styrene by the Ru NPs protected by the dendrons [88].

Besides palladium and platinum, ruthenium nanoparticles were also elaborated. In that case, dendrons having one, two, or four triphenylphosphine functions on their surface were used for milling under air with a mixture of ruthenium trichloride RuCl_3_ and sodium borohydride NaBH_4_ as reducing agent. Ruthenium nanoparticles (Ru NPs) having a diameter in the 2 to 3 nm range and protected by the dendrons were obtained in this way. It should be noted that the largest dendrons afforded the less hindered nanoparticles. Indeed, a smaller number of large dendrons was grafted per Ru NP, compared to the large number of small dendrons, as illustrated in Scheme 12A. These Ru NPs were air-stable upon storage, and all of them efficiently catalyzed hydrogenation of styrene. The best catalytic results were obtained with the largest dendron, which facilitates access of the reagents to the less protected Ru NPs (Scheme 12B) [88].

### 4.3. Synthesis of Titanium Nanoparticles

**Scheme 13:**
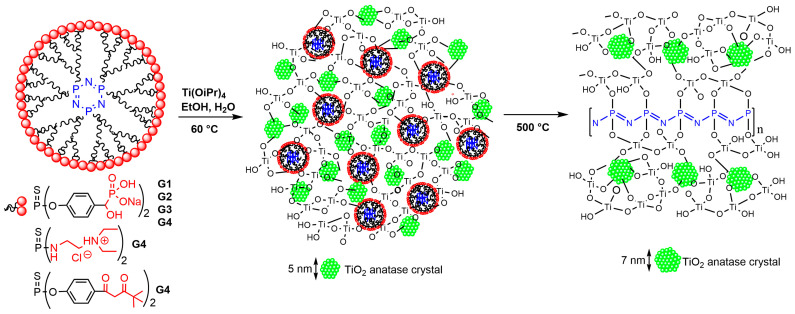
Phosphorhydrazone PPH dendrimers used for the generation of stable anatase nanocrystals, which remained stable even at very high temperature [94].

The possibility of phosphorus dendrimers to react with metal alkoxides was demonstrated early with the elaboration of hybrid materials, from dendrimers with carboxylic acid end groups and either titanium oxo clusters [89] or cerium or titanium alkoxides [90]. On the other hand, a dendron functionalized with phosphonates was able to strongly interact with the surface of nanocrystalline mesoporous titania thin film [91]. Thus, dendrimers of generations 1 to 4, functionalized with phosphonic acids on the surface, were reacted with titanium tetraisopropoxide Ti(OiPr)_4_. Stable porous hybrid materials based on a titanium oxo network entrapping the dendrimers were obtained, which surprisingly included nanocrystals of anatase TiO_2_ (ca., 5 nm). Furthermore, the anatase structure was stable up to 800 °C and did not transform to the brookite or rutile phase, commonly observed at this temperature range [92]. Anatase, rutile, and brookite are three crystalline forms of TiO_2_. The anatase-to-rutile transformation occurs in the temperature range of 700–1000 °C, whereas brookite is converted into rutile by heating to temperatures above 700 °C [93]. Other fourth-generation phosphorus dendrimers equipped with ammoniums or diketone terminal functions were also reacted with Ti(OiPr)_4_ at mild temperature (60 °C) and also afforded materials including anatase nanocrystals (4.8 to 5.2 nm in size). Small-angle X-ray diffraction studies (SAXD) indicated that the material was organized as an ordered mesoporous network. Heating the materials at 500 °C induced the ring opening of the cyclotriphosphazene core of the dendrimers, and the cleavage of the dendrimer branches, to afford a linear polyphosphazene polymer bridged mineral phase, as illustrated in Scheme 13 [94]. The dendritic structure provides a confined medium for the low-temperature crystallization of TiO_2_, and the heteroatoms of the core and the branches (P, N, S) passivate the surface of the anatase nanocrystals. The phase transformation is generally initiated at the anatase surface; thus, its passivation prevents its transformation. Finally, the ring opening polymerization of the cyclophosphazene core restricts the anatase growth and affords thermally stable, interpenetrating mesoporous polyphosphazene–anatase networks [93]. Later on, the materials obtained after calcination at 500 °C were used as catalysts in photocatalytic water splitting for hydrogen production [95]. A recent paper reported the hemolytic activity and cytotoxicity of related dendrimer-TiO_2_ materials [96].

## 5. Conclusions

This review has displayed the usefulness of poly(phosphorhydrazone) PPH dendrimers for both the encapsulation/protection of pre-existing nanoparticles and for the synthesis of nanoparticles, for which the dendrimers act as three-dimensional templates. The presence of heteroelements such as phosphorus, sulfur, and nitrogen in the structure of phosphorhydrazone dendrimers, and also of unsaturated macrocycles in some cases, is particularly suitable for interacting with metals and for the stabilization of metal nanoparticles by passivation of their surface. The generation of the dendrimer is also important, as metal NPs are surrounded by several molecules of small dendrimers, whereas NPs are encapsulated inside large dendrimers. Such a difference generally has an influence on the stability of the NPs, with the encapsulation inside the large dendrimers inducing a better stability. The nature of the terminal functions of the dendrimers can also play an important role in the interactions with the NPs. The role of phosphines or olefinic macrocycles has to be particularly emphasized for their strong associations with metal NPs. Covalent grafting, electrostatic interactions, and π-stacking interactions have all been used to produce dendrimer/NP interactions. Various techniques have been used for the characterization of the dendrimer/NP associations. These methods mainly concerned the characterization of solid matter, in particular by AFM, TEM and HRTEM, SAXRD, WAXRD, EXAFS, EDX, XPS, and IR.

Most of the properties that have been found for these associations of hard nanoparticles (metal) and soft nanoparticles (phosphorhydrazone dendrimers) concerned nanomaterials. The elaboration of multilayers associating dendrimers and metal NPs, and also the fabrication of sensitive chemical or biological sensors, or the stabilization of metal NPs, even at very high temperatures are the main topics displayed in the field of nanomaterials. Several examples in the field of catalysis have been also reported, with emphasis on the efficient recovery and reuse of the associated dendrimers/NPs. The field of biology has not really been investigated up to now, with only two examples concerning the antibacterial properties of silver NPs hosted by a small dendrimer associated with silica NPs, and the hemolytic activity and cytotoxicity of TiO_2_/dendrimers networks. This field certainly needs to be developed, as illustrated by gold nanoparticles encapsulated not in PPH dendrimers but in PAMAM dendrimers functionalized on the surface with β-cyclodextrin, for improved delivery of small interfering RNA (siRNA) to glioblastoma cells [97].

Searching the keywords “dendrimer” and “nanoparticle” generates over 10,000 answers in the Web of Sciences, including over 2000 reviews, in which phosphorhydrazone PPH dendrimers occupy a small but original place. This is due to the unique characteristics of their structure, which includes a large number of heteroatoms (P, S, N), contrarily to all the other types of dendrimers. It should be emphasized that the most cited papers in the field of dendrimers/NPs concern catalysis [18,20] and biology [98], two fields that should be more developed with the phosphorhydrazone dendrimers. 

## Figures and Tables

**Table 1:** MIC and MBC of silica NPs functionalized with pegylated dendrimers hosting silver NPs determined on 4 typical bacterial strains.

Materials	MIC ^1^/MBC ^1^ of *S. aureus* CIP 4.83 (Gram+)	MIC ^1^/MBC ^1^ of *E. hirae* CIP 58.55 (Gram+)	MIC ^1^/MBC ^1^ of *E. coli* CIP 103571 (Gram−)	MIC ^1^/MBC ^1^ of *P. aeruginosa* CIP 104116 (Gram−)
Ag^0^@SidendriPEG	500/>500	250/>500	250/500–1000	250/>500
Ag^0^@SidendriPEG	50–62.5/>500	25–31.25/>500	50–62.5/250	25–31.25/250

^1^ in ppm (silver equivalents).
